# IGFBP-3/IGFBP-3 Receptor System as an Anti-Tumor and Anti-Metastatic Signaling in Cancer

**DOI:** 10.3390/cells9051261

**Published:** 2020-05-20

**Authors:** Qing Cai, Mikhail Dozmorov, Youngman Oh

**Affiliations:** 1 Department of Pathology, Medical College of Virginia Campus, Virginia Commonwealth University, Richmond, VA 23298, USA; qing.cai@vcuhealth.org (Q.C.); mikhail.dozmorov@vcuhealth.org (M.D.); 2 Department of Biostatistics, Massey Cancer Center, Virginia Commonwealth University, Richmond, VA 23298, USA

**Keywords:** IGF system, IGFBP-3, IGFBP-3R, TMEM219, anti-tumor, anti-metastatic, agonists, mAb therapy

## Abstract

Insulin-like growth factor binding protein-3 (IGFBP-3) is a p53 tumor suppressor-regulated protein and a major carrier for IGFs in circulation. Among six high-affinity IGFBPs, which are IGFBP-1 through 6, IGFBP-3 is the most extensively investigated IGFBP species with respect to its IGF/IGF-I receptor (IGF-IR)-independent biological actions beyond its endocrine/paracrine/autocrine role in modulating IGF action in cancer. Disruption of IGFBP-3 at transcriptional and post-translational levels has been implicated in the pathophysiology of many different types of cancer including breast, prostate, and lung cancer. Over the past two decades, a wealth of evidence has revealed both tumor suppressing and tumor promoting effects of IGF/IGF-IR-independent actions of IGFBP-3 depending upon cell types, post-translational modifications, and assay methods. However, IGFBP-3′s anti-tumor function has been well accepted due to identification of functional IGFBP-3-interacting proteins, putative receptors, or crosstalk with other signaling cascades. This review mainly focuses on transmembrane protein 219 (TMEM219), which represents a novel IGFBP-3 receptor mediating antitumor effect of IGFBP-3. Furthermore, this review delineates the potential underlying mechanisms involved and the subsequent biological significance, emphasizing the clinical significance of the IGFBP-3/TMEM219 axis in assessing both the diagnosis and the prognosis of cancer as well as the therapeutic potential of TMEM219 agonists for cancer treatment.

## 1. Introduction

The insulin-like growth factor (IGF) system comprises of ligands IGF-I, IGF-II, its corresponding cell-membrane receptors IGF-I receptor (IGF-IR), IGF-II receptor (IGF-IIR), IGF-binding proteins (IGFBPs), and IGFBP degrading enzymes known as proteases. The IGF system plays a critical role in somatic growth in an endocrine fashion as well as cell proliferation, survival, and differentiation of normal and malignant cells in a paracrine/autocrine fashion. Dysregulation of the IGF system attributes to pathophysiology of a variety of human diseases such as cancer, diabetes, chronic inflammatory disease, and malnutrition. In particular, IGF/IGF-IR-independent actions of IGFBP-3 have been extensively investigated and their involvement in initiation and progression of various cancers has been recognized.

## 2. IGFBP-3

### 2.1. Structure-Function Analysis

**Figure 1:**
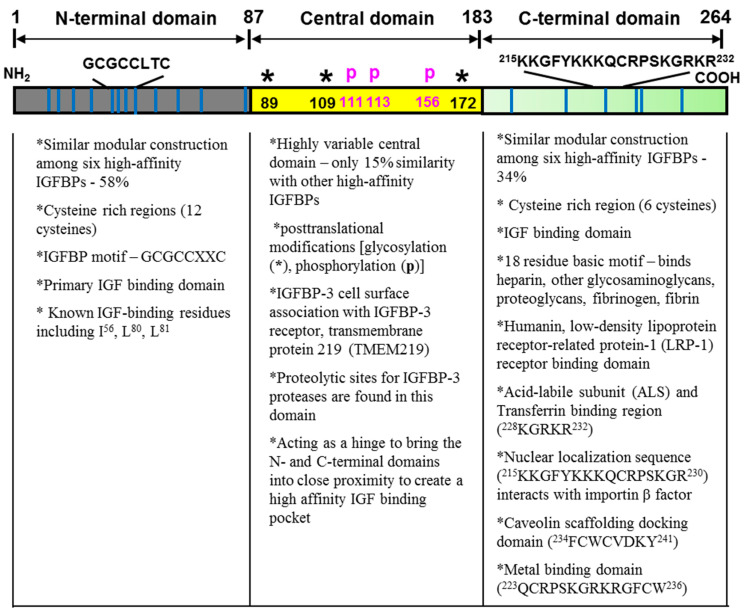
Structure of the mature human IGFBP-3. This figure depicts the three distinct domains of the IGFBP-3 and lists the important functions and motifs/residues within each domain [3]. The vertical blue lines represent 18 cysteine residues in highly conserved *N*-terminal and *C*-terminal domains.

Human IGFBP-3 is comprised of 264 amino acids, of which the molecular mass is 28.7 kDa without any post-translational modifications [1]. The primary structures of human IGFBP-3 consist of three distinct domains: a highly conserved cysteine-rich *N*- and *C*-terminal domains and a nonconserved central domain. Each domain contains various functional motifs/sequences that confer IGFBP-3’s diverse IGF/IGF-IR-dependent and IGF/IGF-IR-independent actions (Figure 1) [2,3,4,5,6]. These distinctive functional motifs/sequences include a caveolin scaffolding docking domain, a metal binding domain, heparin binding motifs, a retinoic acid binding motif, and a nuclear localization sequence.

#### 2.1.1. The Conserved *N*-Terminal Domain

In the mature IGFBP-3 peptide, amino acid residues 1–87 comprise the conserved *N*-terminal domain, which shares approximately 58% similarity with other high-affinity IGFBPs. A well conserved IGFBP motif (GCGCCXXC) present in all IGFBP species is located in this domain. Ten to 12 of the 16–20 cysteines are located in the *N*-terminal domain of high-affinity IGFBPs. Among a total of 18 cysteines in IGFBP-3, 12 cysteines reside in the *N*-terminal domain, which results in the formation of six disulfide bonds within the domain and providing a highly organized tertiary structure. Thus, this conserved *N*-terminal domain shares not only amino acid similarity but also conformational similarities among high-affinity IGFBPs. Important IGF-binding residues including I56, L80, and L81 are also located within this domain [2,3,7].

#### 2.1.2. The Variable Central Domain

The central domain contains 95 amino acids and spans residues 88–183. This domain separates the *N*-terminal domain from the *C*-terminal domain and shares less than 15% similarity with other high-affinity IGFBPs [2]. However, it appears that this domain structurally acts as a hinge between the *N*- and *C*-terminal domains and bring two domains together into close proximity to create a high affinity IGF binding pocket. Post-translational modifications such as glycosylation, phosphorylation, and proteolysis of IGFBP-3 have been found in this domain [8,9,10,11,12]. The functional significance of those post-translational modifications has been reported that glycosylation can affect cell interactions, that phosphorylation can affect IGF-binding affinity and susceptibility to proteases, and that proteolysis can affect both IGF-dependent and IGF-independent actions [4,11,12,13]. Three *N*-linked glycosylation sites at asparagine 89, 109, and 172, and phosphorylation sites at serine 111, 113, 156, 165, and at threonine 170, as well as proteolytic sites for metalloproteases (MMPs) and serine proteases exist in this domain [8,9,10,12,13]. The central domain is responsible for the interaction with the IGFBP-3 specific receptor known as transmembrane protein 219 (TMEM219) [14,15].

#### 2.1.3. The Conserved *C*-Terminal Domain

This domain spans residues 184–264, containing six cysteines with three disulfide bonds. This domain is also important in IGF binding [16,17,18,19]. Since the IGFBP-3 fragment that contains only *N*- or *C*-terminal domains has significantly reduced affinity for IGFs, it requires an IGF-binding pocket involving both domains for high affinity binding to IGFs. Several functionally important sequences/motifs are present in this domain such as binding for heparin, glycosaminoglycans, proteoglycans, fibronectin, fibrin, transferrin, plasminogen, acid-labile subunit (ALS), and metals such as iron, zinc, and nickel [3,4,5,20,21,22,23,24]. Both IGFBP-3 and IGFBP-3-IGF complexes bind fibrinogen, fibrin, and plasminogen. Furthermore, a nuclear localization sequence (NLS) [25] and a caveolin-scaffolding domain consensus sequence [26] also reside in this domain.

### 2.2. IGF/IGF-IR Dependent Actions of IGFBP-3

The principal action of IGFBP-3 is to transport IGF-I and IGF-II in circulation, and, thereby, prolong the half-life of IGFs. IGFBP-3 has a higher affinity for IGFs (Kd approximately 10^−10^ M) than their respective receptors. In serum, most of the IGFs circulate as a 150 kDa complex, consisting of 7·5 kDa IGF-I or IGF-II and 45 kDa glycosylated IGFBP-3 and 90 kDa ALS [3,4,5,27,28,29]. The biological activity of circulating IGFs in the tissues is determined by the transition of IGF from 150 kDa complex to the 55 kDa IGF-IGFBP-3 complex and subsequent proteolysis of the complex to release IGF in the circulation or in the local body fluid. In addition to functioning as an IGF transporter, IGFBP-3 also functions as modulators of IGF availability and activity at the cellular levels in an autocrine or paracrine manner [25,28,29,30,31,32].

IGFBP-3 can inhibit or enhance IGF actions, depending on cell types, the cellular environment, IGFBP-3 concentration, and post-translational modifications such as glycosylation, proteolysis, and phosphorylation [4,11,12,13]. IGFBP-3 has shown to inhibit IGF activity by competitively binding IGFs and preventing its binding to IGFRs [29,30]. On the other hand, IGFBP-3 can enhance IGF activity by increasing IGF concentration in the extracellular microenvironment by binding to heparin and proteoglycans, and, thereby, acting as a reservoir of IGFs [20,21,22].

### 2.3. IGF/IGF-IR Independent Actions of IGFBP-3

The IGF/IGF-IR-independent actions of IGFBP-3 have been shown to contribute to the pathophysiology of various human diseases such as cancer, diabetes, obesity, fatty liver disease, ischemia, and Alzheimer’s disease [15,33,34,35,36,37,38,39,40,41,42,43,44,45,46,47]. In an early era of IGFBP-3 research in cancer, many studies demonstrated that IGFBP-3 is upregulated by different types of cell growth inhibitors at the transcriptional level in a variety of human cancer cells. These include anti-estrogens (Tamoxifen, ICI-182780), TGF-β, retinoic acid, TNF-α, vitamin D, histone deacetylase inhibitor sodium butyrate, and anti-cancer dietary components including silibinin, apigenin, lycopene, resveratrol, curcumin, and quercetin [48,49,50,51,52,53,54,55,56,57]. In particular, the tumor suppressor gene p53 has been shown to upregulate IGFBP-3 at the transcriptional level [58,59]. Two p53 binding sites, Box A and Box B, were identified in the first and second introns of the IGFBP-3 gene, based on homology to the p53 binding consensus sequence [58]. Further studies using p53 mutants have revealed a link between p53′s activation of IGFBP-3 transcription and its induction of apoptosis by showing that the mutants that lost the ability to activate IGFBP-3 could not induce apoptosis [60]. Further research also demonstrated that the transfection of doxycycline-inducible p53 plasmids resulted in increased expression of p53 and IGFBP-3 and, subsequently, induced apoptosis in p53-negative PC-3 prostate cancer cells [40]. This p53-depedent induction of apoptosis was inhibited by treating with IGF-I, IGFBP-3 blocking antibodies, and IGFBP-3 antisense oligonucleotides, which demonstrated p53-dependent IGFBP-3′s proapoptotic function. In light of p53 dependency of IGFBP-3 expression, ΔNp63α, an isoform of tumor suppressor p63 with both dominant negative (ΔN) activities and a potent repressor of p53-mediated transactivation has been demonstrated to suppress expression of IGFBP-3 [61]. It appears that ΔNp63α binds the p53 binding sites, Box A and Box B, in the IGFBP-3 gene, and, thereby, inhibits p53-dependent IGFBP-3 expression and presumably suppresses IGFBP-3-induced apoptosis. However, evidence also supports that IGFBP-3 can be induced in a p53-independet manner [40]. Treatment with genotoxic drugs such as etoposide and Adriamycin resulted in increased IGFBP-3 expression in p53-negative PC-3 prostate cancer cells.

Moreover, several studies demonstrated that the loss of IGFBP-3 expression by DNA methylation is linked to tumorigenesis and cancer progression as well as intrinsic and/or acquired resistance to radiotherapy and chemo-drugs such as cisplatin in many different types of cancer including lung, colon, and ovarian cancers [62,63,64,65,66,67,68,69]. These findings strongly suggested that IGFBP-3 may exert anti-proliferative and anti-tumor functions beyond its ability to modulate IGF functions (IGF/IGF-IR dependent actions), but the underlying mechanisms involved remain largely unknown. Since then, there has been an intensive investigation toward characterizing the molecular and cellular mechanisms for IGF/IGF-IR-independent antitumor effects of IGFBP-3 in human cancer in vitro and in vivo. It is clear that IGFBP-3 exerts its IGF/IGF-IR-independent biological actions through interactions with a variety of binding partners on cell surfaces and within cells.

#### 2.3.1. IGFBP-3 Binding Partners on the Cell Surface

The very first evidence for the IGF/IGF-IR-independent actions of IGFBP-3 was the identification of specific cell surface binding between IGFBP-3 and cell surface proteins and subsequent cell growth inhibition in Hs578T human triple negative breast cancer (TNBC) cells [33,70]. These initial findings demonstrated that only IGFBP-3 specifically binds to the cell surface among IGFBPs and the central domain of IGFBP-3 is necessary for the binding. IGFs attenuated the cell surface binding and the subsequent growth inhibitory effects of IGFBP-3 by forming IGF-IGFBP-3 complexes. The existence of high-affinity binding sites for IGFBP-3, which is typical of receptor-ligand interactions, were found. The binding sites further demonstrated 20- and 28-kDa cell surface proteins as putative receptors. Based on biochemical and functional characteristics, these proteins are later proven to be an IGFBP-3 receptor, TMEM219, which was identified by a yeast two-hybrid screening using the central domain of IGFBP-3 from the same Hs578T human TNBC cell line [15]. This IGFBP-3 receptor will be further discussed in Section 3.

At present, a few proteins have been identified as IGFBP-3 cell surface binding partners such as the low-density lipoprotein receptor-related protein-1 (LRP-1)/α2M receptor [71], autocrine motility factor (AMF)/phosphoglucose isomerase (PGI) [72], latent TGF-β binding protein-1 (LTBP-1), caveolin, and transferrin/transferrin receptor [26,73]. The LRP-1/α2M receptor, also known as TGF-β type V receptor, is shown to mediate IGFBP-3-induced cell growth inhibition independent of IGF [74,75]. In addition, it plays a crucial role for cellular internalization of IGFBP-3 since LRP knock-out cells exhibited significant reduction of IGFBP-3 internalization when compared with LRP-expressing mouse embryonic fibroblasts [76]. While AMF/PGI, which is a tumor-secreted cytokine, is endocytosed and regulates cell migration, proliferation, and survival, IGFBP-3 has been shown to inhibit AMF/PGI-induced cell migration in T47D and MCF-7 breast cancer cells [72]. LTBP-1, which is a component of the latent TGF-β complex and a part of structural component of the ECM, is involved in sequestration of latent TGF-β in the ECM and delivery of TGF-β to the plasma membrane [77]. Although the functional significance of IGFBP-3 binding to LTBP-1 as well as the large latent complex has not been fully elucidated, it may be a potential mechanism whereby IGFBP-3 can interact with the TGF-β system [78]. Since a substantial amount of LTBP-1 can be secreted by cells without bound TGF-β, IGFBP-3 may also involve TGF-β-independent functions of LTBP-1 [79].

IGFBP-3 interaction with caveolin-1 through a caveolin-scaffolding sequence induced IGFBP-3 internalization [26]. Furthermore, recent research indicated that caveolin-1 is an oncogenic membrane protein and is associated with endocytosis, extracellular matrix organization, cholesterol distribution, cell migration, and signaling. This strongly suggests the potential regulation of IGFBP-3 on these caveolin-1-induced functions [80]. IGFBP-3 also binds to transferrin and forms an IGFBP-3-tranferrin-transferrin receptor complex, providing another mechanism for IGFBP-3 internalization and signaling [26,73]. IGFBP-3 internalization was inhibited by co-incubation and extracellular sequestration with IGF-I, and was dependent on the transferrin-binding *C*-terminal peptide region of IGFBP-3 [26]. By the same token, blocking transferrin receptor-mediated endocytosis suppressed IGFBP-3 internalization and IGFBP-3-induced apoptosis [26]. At present, it remains unclear whether TMEM219 is a sole IGFBP-3 receptor mediating IGF/IGF-IR independent antitumor actions of IGFBP-3 or whether the previously mentioned cell surface binding partners are also partly involved in IGFBP-3 internalization and subsequent IGF/IGF-IR independent actions in cytoplasmic and nuclear compartments.

#### 2.3.2. IGFBP-3 Binding Partners within Cells

Although IGFBP-3 can be internalized to the cytoplasmic compartment and translocated to the nucleus through the NLS in the conserved *C*-terminal domain, limited knowledge is available on whether nuclear targeting of IGFBP-3 occurs in all types of cells or requires specific cellular conditions. Nevertheless, IGFBP-3 has been shown to interact with cytoplasmic/nuclear proteins. These include humanin [37], RNA polymerase II binding subunit 3 (Rpb3) [81], GalNAc-T14 [82], glucose-regulated protein 78 (GRP78) [83,84], nuclear retinoid X receptor (RXR) [85], retinoic acid receptor (RAR) [86], Nur77 [87], vitamin D receptor (VDR) [88], and peroxisome proliferator-activated receptor-γ (PPARγ) [89].

Humanin is a mitochondrial-derived peptide that inhibits neuronal cell death induced by mutant genes in Alzheimer’s disease [37]. Humanin has been shown to bind to IGFBP-3 and inhibit nuclear translocation and induction of apoptosis of IGFBP-3 in human lung cancer cells by suppressing the IGFBP-3 interaction with importin-β [90]. Rpb3, which is an essential component of the mRNA transcription apparatus, aids the recruitment of the polymerase complex to specific transcription factors. Rpb3 has been shown to interact with the NLS motif of IGFBP-3 and might lead to IGFBP-3′s role in modulating gene transcription [81]. GalNAc-T14, a large subfamily of glycosyltransferases residing in the Golgi complex, catalyze the first step in the *O*-glycosylation of mammalian proteins by transferring *N*-acetyl-d-galactosamine (GalNAc) to peptide substrates [91]. Since GalNAc-T14 has been shown to be associated with poor recurrence-free survival and promote cell migration and invasion as well as metastasis through the Wnt signaling in lung cancer [92], IGFBP-3 may interfere with pro-tumorigenic and pro-metastatic GalNAc-T14 signaling by complexing with GalNAc-T14 in certain types of cancer including lung cancer. On the other hand, GalNAc-T14 has been also shown to inhibit IGFBP-3-induced cell proliferation and colony formation in glioblastoma cells. Although overexpression of IGFBP-3 induced expression of Cyclin E, CDK2, and p-ERK1/2, and overexpression of GalNAc-T14 inhibited these IGFBP-3 effects in glioblastoma cells, no evidence was presented whether direct binding of these two proteins is involved in observed biological outcomes [93].

GRP78, which is also known as immunoglobulin heavy-chain binding immunoglobulin protein (BiP), plays a critical role for endoplasmic reticulum integrity and stress-induced autophagy in mammalian cells [94]. When unfolded or misfolded proteins accumulate in the ER (called ER stress), an unfolded protein response (UPR) is activated through the induction of GRP78 as the first defense response, which, thereby, restores normal function of the ER by attenuating global translation and increasing the folding capacity of the ER [95]. It has been shown that the elevated expression of GRP78 is correlated with cancer malignancy, metastasis, and drug resistance in a variety of cancers, including breast cancer, prostate cancer, lung cancer, and glioma [96]. In line with these findings, GRP78 has been further shown to possess pro-survival and anti-apoptotic properties [97]. Interaction of IGFBP-3 and GRP78 has been identified in human breast cancer cells using a yeast two-hybrid screening [83]. In this study, overexpression of IGFBP-3 showed that IGFBP-3 binding to GRP78 results in the disruption of the GRP78-caspase-7 complex, which, thereby, activates caspase-7, and, subsequently, induces apoptosis in anti-estrogen-resistant breast cancer cells. These findings strongly suggest that IGFBP-3 could sensitize anti-estrogen-resistant breast cancer cells to anti-estrogen such as ICI 182,780 by preventing the anti-apoptotic function of GRP78. On the contrary, IGFBP-3 has been shown to enhance the survival of cells subjected to glucose starvation and hypoxia by inducing autophagy in a GRP78-dependent manner in human breast cancer cells, which suggests that IGFBP-3 may play a key role in mediating an autophagic survival response [84]. Although the biological outcomes of IGFBP-3 are much different depending on the cellular environment, it is clear that the specific interaction of GRP78 and IGFBP-3 is attributed to the observed IGFBP-3 effects.

IGFBP-3 has also been shown to inhibit cell growth and induce apoptosis through an interaction with nuclear proteins such as retinoid X receptor (RXR)-α, retinoic acid receptor (RAR), and Nur77 [98]. RXR is involved in physiological functions of thyroid hormone, steroid hormones, embryonic development, apoptosis, and homeostasis [99,100,101]. RXR heterodimerizes with Nur77, a nuclear receptor transcription factor, and, thereby, enhances its DNA binding ability and regulates apoptosis in various cancers [102]. IGFBP-3 binds RXR-α and RAR and, subsequently, modulates RAR/RXR and RXR/Nur77 signaling, which, thereby, induces apoptosis [81,82]. It has been further shown that Nur77 translocates to the nucleus and initiates apoptosis in the presence of IGFBP-3 [98]. However, recent studies also showed that IGFBP-3 mutants that failed to translocate to the nucleus and lost binding ability to RXR-α, still induced apoptosis in breast cancer cells [103,104]. This suggests that IGFBP-3 may either utilize multiple mechanisms for its anti-tumor actions depending upon the cellular environment or the observed IGFBP-3 interaction with cytoplasmic/nuclear partners may not represent major IGFBP-3 anti-tumor signaling. It is clear that IGFBP-3 exerts a pro-apoptotic and anti-proliferative IGF/IGF-IR independent actions through multiple mechanisms such as an interaction with the IGFBP-3 receptor and other binding partners on the cell surface and within cells as well as nuclear association. The remainder of this review will focus on the IGFBP-3 receptor TMEM219 in human cancer by mainly providing the evidence to date regarding the IGFBP-3/TMEM219 system as an anti-tumor and anti-metastatic signaling in human cancer.

## 3. TMEM219 as an IGFBP-3 Specific Receptor

Early studies in the IGF/IGF-IR-independent actions of IGFBP-3 have been shown that IGFBP-3 binding to a cell surface protein is required for its anti-proliferative action in human breast cancer cells and that the central domain of IGFBP-3, which is the least conserved region among IGFBPs 1–6, is responsible for cell surface binding [33,70,105]. Furthermore, IGFBP-3 has been shown to induce apoptosis by activating caspase-8 cleavage, but not cytochrome c release or caspase-9 cleavage involved in the death receptor-mediated apoptotic pathways in MCF-7 breast cancer cells [106]. These findings strongly suggested the existence of an IGFBP-3-specific receptor mediating the direct anti-proliferative and pro-apoptotic effects of IGFBP-3 in a variety of cancer cells.

**Figure 2:**
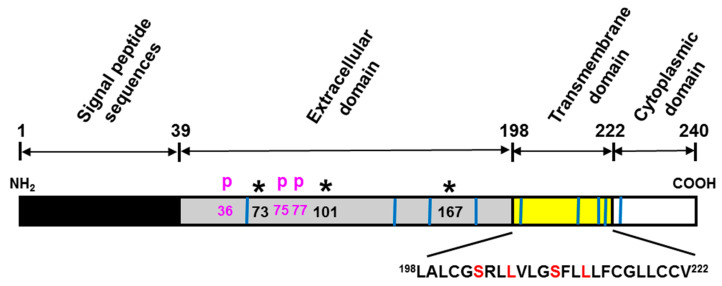
Structure of human IGFBP-3R (TMEM219). The 202-residue mature IGFBP-3R, omitting the 38-residue signal peptide, is comprised of three domains: extracellular, transmembrane, and cytoplasmic domains. Extracellular domain contains three potential *N*-glycosylation sites (residues 73, 101, 167) and three potential phosphorylation sites (S36, T75, T77). The single-span transmembrane domain contains a leucine zipper-like heptad repeat pattern characteristic of leucine zipper interaction domains. The letters in red correspond to a-type and d-type interfacial residues in leucine zipper interaction domains [15]. The vertical blue lines represent nine cysteine residues.

As an effort to identify a novel cell death receptor specific for IGFBP-3, yeast two-hybrid screening was employed using a cDNA construct encoding amino acid residues 88–148 of the variable central domain of IGFBP-3 as bait against an Hs578T human TNBC cell cDNA library. As a result, a functionally unknown transmembrane protein TMEM219 has been identified as an IGFBP-3 specific interacting protein and later designated as an IGFBP-3 receptor (IGFBP-3R) [15]. TMEM219 consists of four exons comprising the 915-base pair cDNA sequence on chromosome 16q13 and represents a 240-amino acid polypeptide. Further analysis of the deduced amino acid sequence indicated that the 202-residue mature human IGFBP-3R consists of an extracellular domain, a putative single-span transmembrane domain, and a short *C*-terminal cytoplasmic domain (Figure 2). The extracellular domain contains three potential *N*-glycosylation sites and three phosphorylation sites. The transmembrane domain contains a leucine zipper-like heptad repeat pattern of amino acids that appear to involve dimerization/oligomerization of the membrane proteins. This very unique leucine zipper sequence is also present in the single-span transmembrane domain of the erythropoietin receptor and the discoidin domain receptor [106,107]. Additionally, IGFBP-3R activates caspase-8-induced apoptosis in unconventional ways: (1) IGFBP-3R and inactive procaspase-8 is pre-complexed at the resting stage, and IGFBP-3 binding to IGFBP-3R releases procaspase-8, and, thereby, activates caspase-8-dependent apoptosis, and (2) IGFBP-3R complexes with procasepase-8 without involvement of a typical death domain (DD) sequence. The DD sequence in the intracellular portion of the receptor is required to form a death-inducing signaling complex (DISC) by recruiting adaptor proteins (FADD) and procaspase-8 after receptor activation in various death receptors such as the TNF-α receptor, TNF-related apoptosis-inducing ligand receptor 1 (TRAIL-R1/DR4), TRAIL-R2 (APO-2/DR5), and CD95 (Fas, APO-1). However, similar to IGFBP-3R, a few other proteins have been shown to interact with caspase-8 and induce apoptosis despite the lack of a DD sequence [108,109]. IGFBP-3R is located in both the plasma membrane and cytoplasm, but not in the nucleus of the cancer cells. This cell surface IGFBP-3R interacts specifically with IGFBP-3 but not with other high-affinity IGFBPs, activates procaspase-8, and mediates IGFBP-3-induced apoptosis in many different types of cancer cells and tumor suppression in both prostate and breast cancer xenograft mouse models. Further knockdown of IGFBP-3R attenuates IGFBP-3-induced caspase activities and apoptosis, whereas its overexpression elicited the opposite effects [15,65,110,111]. These findings clearly indicate that IGFBP-3R (TMEM219) is a bona fide IGFBP-3 receptor and mediates anti-tumor activities of IGFBP-3.

In addition, IGFBP-3 has been shown to suppress tumor-induced NF-κB activity via activation of caspase-8 and caspase-3/7 in an IGF/IGF-IR-dependent manner in prostate cancer cells [111]. IGFBP-3 suppresses NF-κB activity in a unique way. It exerts caspase-induced degradation of IκBα and NF-κB, but not other components such as IKK. IGFBP-3 also inhibited the expression of NF-κB-regulated factors such as VEGF, IL-8, ICAM-1, and VCAM-1. This inhibitory action of IGFBP-3 was IGF/IGF-IR-independent since the IGFBP-3 mutant devoid of IGF binding affinity had a similar inhibitory effect. Furthermore, IGFBP-3R has been shown to be responsible for IGFBP-3-induced suppression of NF-κB activity in cancer cells. These findings indicate that IGFBP-3 in addition to inducing apoptosis, also suppresses tumor-induced NF-κB activity, and, thereby, enhances the inhibition of tumor growth, angiogenesis, invasion, metastasis, and chemoresistance [111].

Recent reports further explored the therapeutic potential of the IGFBP-3/IGFBP-3R axis in cancer by developing an IGFBP-3R agonistic monoclonal antibody (mAb) [112]. It has been shown that activation of IGFBP-3R by IGFBP-3 and IGFBP-3R agonistic mAb inhibits cell growth by inducing apoptosis and by tumor-induced NF-κB activity specifically in cancer cells, but not in normal cells. At present, for the cancer cell, specific pro-apoptotic properties of IGFBP-3 and IGFBP-3R agonistic mAb are not fully elucidated. However, a few potential mechanisms can be speculated based on the findings in tumor-specific targeting of the death receptor (DR)-4 and DR-5 agonistic mAb therapy despite the presence of DR-4/DR-5 in normal cells [113,114,115]. These include: (1) decreased level of the cell surface DR-4/DR-5 in normal cells compared to cancer cells, (2) differential expression of unknown intracellular inhibitor(s) of apoptosis downstream of caspase-8, and (3) changes in apoptotic potency due to different glycosylation patterns of DRs.

In addition, IGFBP-3R agonistic mAb lost anti-proliferative effects in IGFBP-3R knockout cells. These in vitro data indicate that IGFBP-3R is indispensable for anti-tumor functions of IGFBP-3 and IGFBP-3R agonistic mAb in a variety of cancer cells. Further anti-tumor and anti-metastatic effects of IGFBP-3R agonistic mAb have been shown in vivo using MDA231 TNBC and patient-derived TNBC xenograft models [112]. Taken together, these findings provide evidence that IGFBP-3R (TMEM219) is a bona fide IGFBP-3 receptor and a potential target for cancer therapy.

## 4. Clinical Insights of IGFBP-3/IGFBP-3R (TMEM219) System in Cancer

Although IGFBP-3 may utilize multiple mechanisms for its anti-tumor actions, current findings suggest that the IGFBP-3/IGFBP-3R axis may constitute a novel anti-tumor/anti-metastatic signaling pathway and a novel potential therapeutic target in cancer. However, since limited knowledge is available on clinicopathologic significance and prognostic value of the IGFBP-3/IGFBP-3R system, the remainder of this review will focus on its clinical significance using data mining and analyses of publicly available databases including The Cell Index (CELLX) database (http://cellx.sourceforge.net) and The Cancer Genome Atlas (TCGA) database (https://portal.gdc.cancer.gov). Log2-transformed RSEM (RNA-Seq by Expectation-Maximization) [116] gene expression values were obtained.

### 4.1. IGFBP-3 and TMEM219 Gene Expression in Tumor and Normal Samples

**Figure 3:**
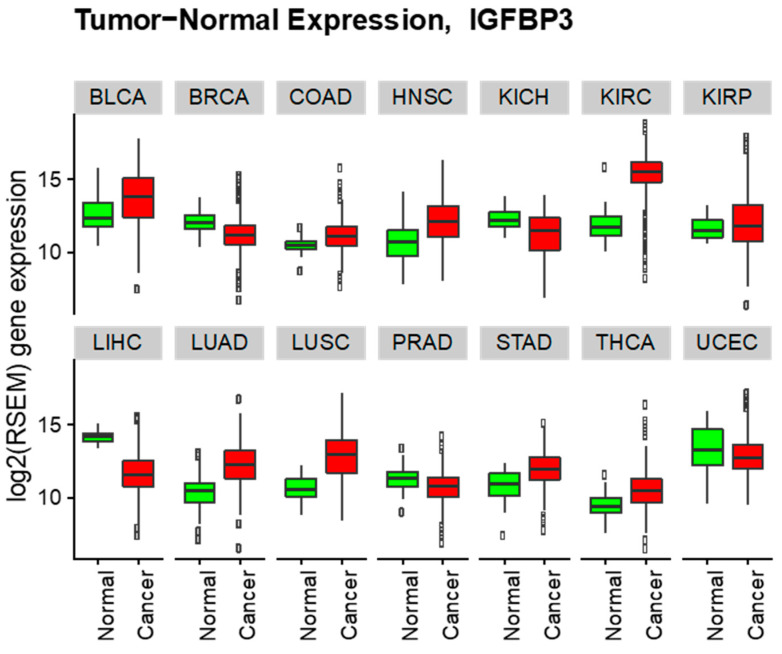
Cancer vs. normal IGFBP-3 log2 RSEM gene expression boxplots (all cancer). Description of abbreviations presented in Table 1.

Analysis of IGFBP-3 expression identified highly variable expression levels among different types of cancer as well as normal tissues (Figure 3). Further differential expression of IGFBP-3 was analyzed in cancers and the counterpart normal tissues where at least 12 normal samples were available (Table 1).

Increased IGFBP-3 expression was observed in kidney renal clear cell carcinoma (log2 fold change +3.44), lung squamous cell carcinoma (log2 fold change +2.26), lung adenocarcinoma (log2 fold change +1.94), head and neck squamous cell carcinoma (log2 fold change +1.32), stomach adenocarcinoma (log2 fold change +1.18), thyroid carcinoma (log2 fold change +1.11), bladder urothelial carcinoma (log2 fold change +1.05), colon adenocarcinoma (log2 fold change +0.65), and kidney renal papillary cell carcinoma (log2 fold change +0.34). On the other hand, decreased expression of IGFBP-3 was observed in liver hepatocellular carcinoma (log2 fold change −2.53), kidney chromophobe (log2 fold change −1.07), breast invasive carcinoma (log2 fold change −0.82), prostate adenocarcinoma (log2 fold change −0.56), and uterine corpus endometrial carcinoma (log2 fold change −0.43).

**Figure 4:**
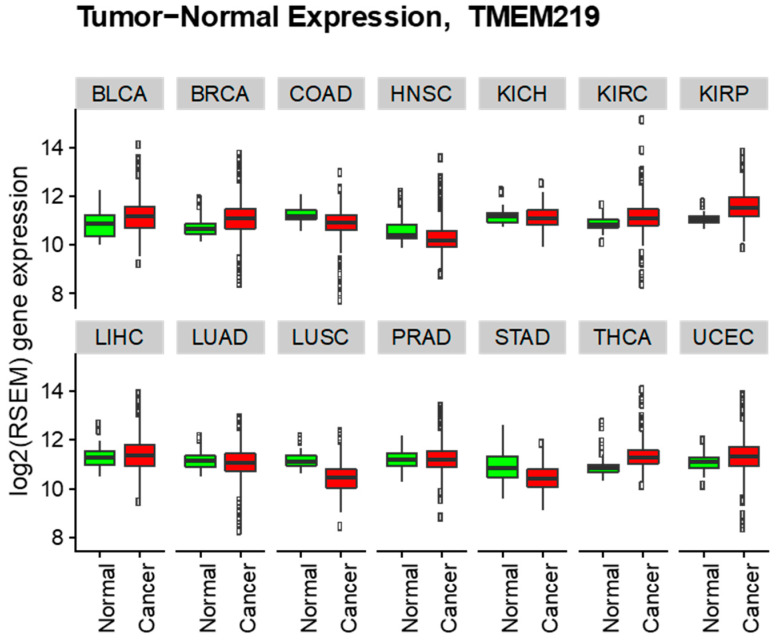
Cancer vs. normal TMEM219 log2 RSEM gene expression boxplots (all cancer). Description of abbreviations presented in Table 2.

In the same datasets, TMEM219 expression also showed variable expression patterns, but had less variation when compared to IGFBP-3 among different types of cancer as well as normal tissues (Figure 4). Analysis of differential expression of TMEM219 revealed significant increased TMEM219 expression in 6 out of 14 tumors (Table 2). These include kidney renal papillary cell carcinoma (log2 fold change +0.53), thyroid carcinoma (log2 fold change +0.40), breast invasive carcinoma (log2 fold change +0.39), kidney renal clear cell carcinoma (log2 fold change +0.29), bladder urothelial carcinoma (log2 fold change +0.27), and uterine corpus endometrial carcinoma (log2 fold change +0.22). On the contrary, decreased TMEM219 expression was observed in lung squamous cell carcinoma (log2 fold change −0.74), stomach adenocarcinoma (log2 fold change −0.42), colon adenocarcinoma (log2 fold change −0.36), head and neck squamous cell carcinoma (log2 fold change −0.32), lung adenocarcinoma (log2 fold change −0.12), and kidney chromophobe (log2 fold change −0.10). In summary, these results suggest IGFBP-3 as a diagnostic biomarker and TMEM219 as a therapeutic target in certain types of tumors. Importantly, TMEM219 agonists may represent a novel therapy for tumors with significantly lower expression of IGFBP-3 but not TMEM219 compared to the counterpart normal tissues, such as breast invasive carcinoma, uterine corpus endometrial carcinoma, liver hepatocellular carcinoma, and prostate adenocarcinoma.

### 4.2. Pan-Cancer Survival Effect of IGFBP-3 and TMEM219

**Figure 5:**
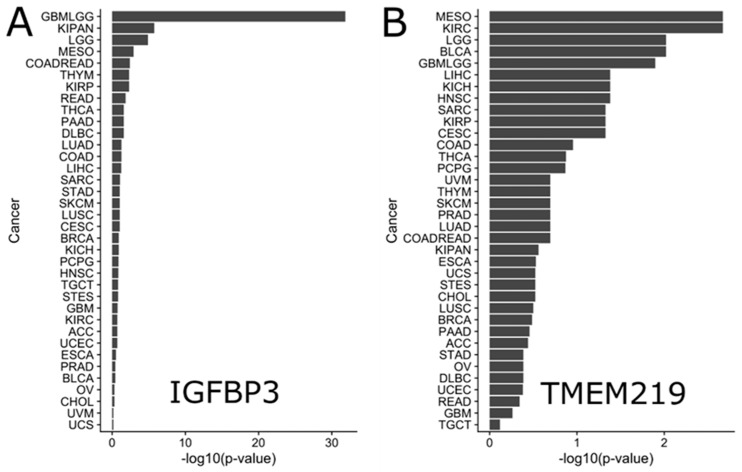
Survival effect of IGFBP-3 (**A**) and TMEM219 (**B**) in various cancers. Larger size of each bar corresponds to the more significant effect on survival.

To investigate the effect of IGFBP-3 and TMEM219 expression on survival in clinical settings, the RNA-seq data from TCGA was analyzed (Figure 5). Gene expression data summarized as RSEM values were obtained using the TCGA2STAT R package v.1.2, along with the corresponding clinical annotations. Data for each of the 34 cancers were obtained separately. The data were log2-transformed and analyzed using Kaplan-Meier curves and the Cox proportional hazard model. Each gene of interest was analyzed for its effect on survival by separating patients into high/low expression subgroups. The scanning approach KaplanScan, used on the R2 Genomics web portal [117], was used to estimate the best gene expression cutoff that separates high/low expression subgroups with differential survival (R code modified from Reference [118]). In addition to survival analysis across all cancers, further survival analysis was performed within clinical subgroups of specific cancers, e.g., in “race-black or African-American” subgroup. *p*-values were corrected for multiple testing using the Benjamin-Hochberg (FDR) method [119] and reported throughout unless noted otherwise. Only subgroups with >40 patients were considered. This approach allowed us to understand the effect of IGFBP3 and TMEM219 expression on the level of individual cancers and in specific population subgroups.

**Figure 6:**
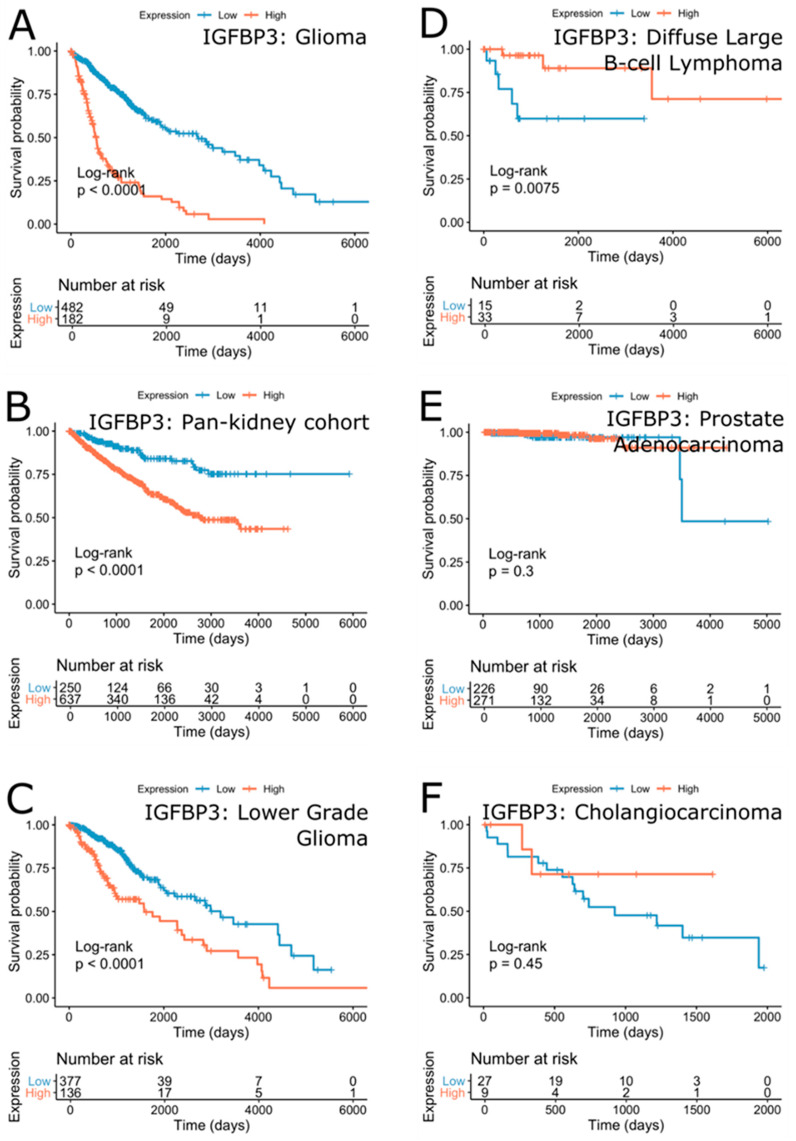
Kaplan-Meier plots of top cancers with survival outcomes separated by the expression of IGFBP-3 with a negative correlation (**A**–**C**) and a positive correlation (**D**–**F**). Unadjusted *p*-values are shown.

Analysis of the survival effect of IGFBP-3 expression in all cancers identified its highly significant effect on survival in glioma (FDR = 1.51∙10^−32^ (Hazard Ratio, HR = 4.39)) (Figure 5A and Figure 6A). Survival in pan-kidney cohort (KICH+KIRC+KIRP), lower grade glioma, mesothelioma, colorectal adenocarcinoma was similarly affected by IGFBP-3 to a lesser extent (FDR = 1.74∙10^−6^ (HR = 2.73), 1.25∙10^−5^ (HR = 2.36), 1.22∙10^−3^ (HR = 2.98), 3.87∙10^−3^ (HR = 2.20), respectively) (Figure 6B,C). On the other hand, higher IGFBP-3 was suggestive of better survival outcome in lymphoid neoplasm diffuse large B-cell lymphoma (FDR = 2.58∙10^−2^ (HR = 0.14)), breast cancer (1.20∙10^−1^ (HR = 0.74)), prostate adenocarcinoma (3.43∙10^−1^ (HR = 0.51)), cholangiocarcinoma (4.84∙10^−1^ (HR = 0.57)), bladder urothelial carcinoma (3.63∙10^−1^ (HR = 0.84)), and uterine carcinosarcoma (6.65∙10^−1^ (HR = 0.860)) (Figure 6D–F).

In summary, these results suggest IGFBP-3 as a prognostic biomarker in glioma, mesothelioma, kidney, and colorectal cancers with lower expression suggestive of better survival outcome, whereas diffuse large B-cell lymphoma, cholangiocarcinoma, bladder urothelial carcinoma, uterine carcinosarcoma, breast, and prostate cancer with higher expression suggestive of better survival outcome. Of note, the observed dichotomy of IGFBP-3 expression and patients’ survival in various cancers may be attributed to other factors such as IGF-1/IGF-2 expression, IGFBP-3 polymorphism status, tumor suppressor p53 family status, tumor metabolic characteristics, and others. In addition, functional IGFBP-3 protein levels in circulation or in tumor and ratio of IGF-I and IGFBP-3 in circulation should be further factored to interpret the TCGA data.

**Figure 7:**
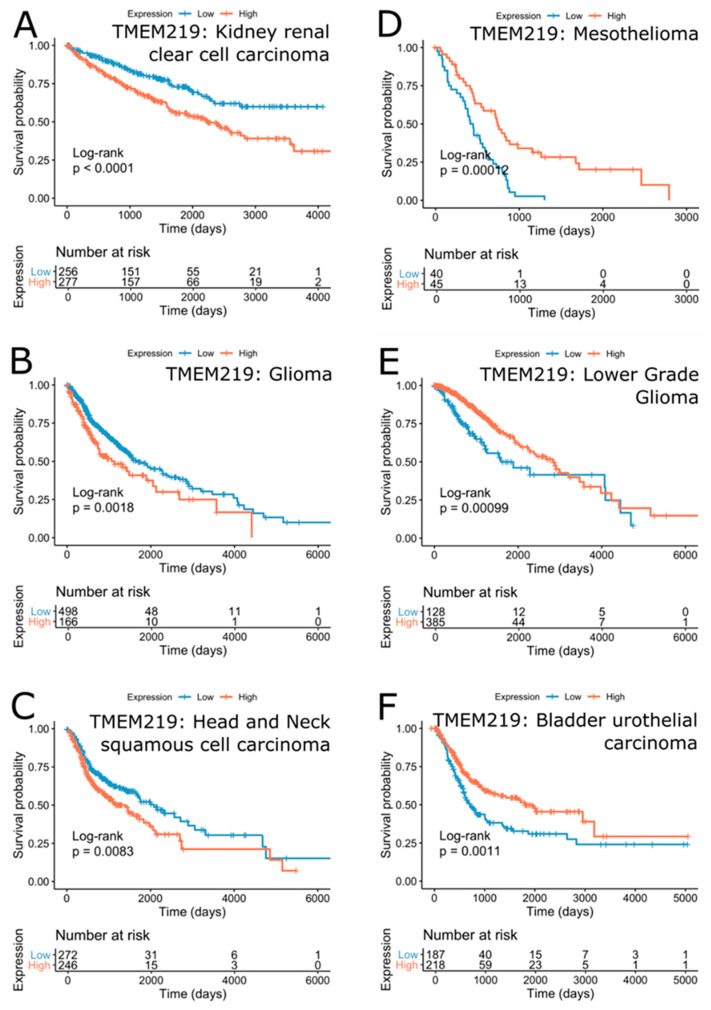
Kaplan-Meier plots of top cancers with survival outcomes separated by the expression of TMEM219 with a negative correlation (**A**–**C**) and a positive correlation (**D**–**F**). Unadjusted *p*-values are shown.

The TMEM219 survival effect was less significant than that of IGFBP-3 (Figure 5B). Nevertheless, the lower expression of the TMEM219 gene was associated with survival in kidney renal clear cell carcinoma (FDR = 2.14∙10^−3^ (HR = 1.85)), glioma (FDR = 1.27∙10^−2^ (HR = 1.56)), lymphoid neoplasm diffuse large B-cell lymphoma (FDR = 4.110.54)), head and neck squamous cell carcinoma (FDR = 4.17E-2 (HR = 1.43)) and pan-kidney cohort (KICH+KIRC+KIRP) (FDR = 2.76E-1 (HR = 1.2)) (Figure 7A–C). On the contrary, the higher expression of TMEM219 was better for survival in mesothelioma (FDR = 2.14E-3 (HR = 0.39)), lower grade glioma (FDR = 9.54E-3 (HR =0.54)), prostate adenocarcinoma (FDR = 2.02∙10^−1^ (HR = 0.18)), thyroid carcinoma (FDR = 1.332∙10^−1^ (HR = 0.39)) and bladder urothelial carcinoma (FDR = 9.54∙10^−3^ (HR = 0.61)) (Figure 7D–F).

**Figure 8:**
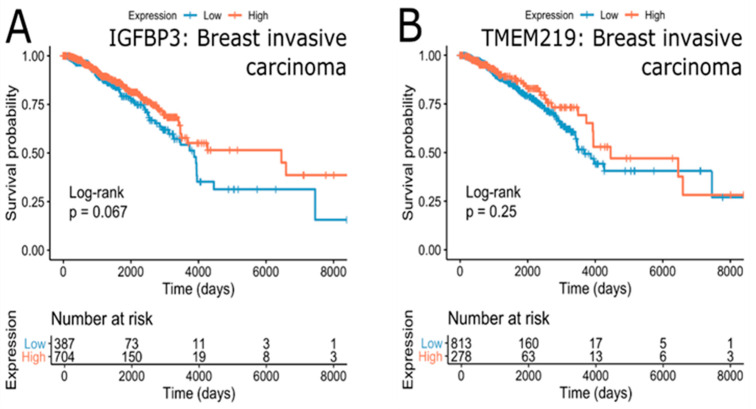
Kaplan-Meier plots of breast cancer cohort with survival outcomes separated by the expression of IGFBP-3 (**A**) and TMEM219 (**B**). Unadjusted *p*-values are shown.

In addition to the cancer types expected to be affected by the IGFBP-3/TMEM219 system, bladder urothelial carcinoma and head and neck squamous cell carcinoma appear to be significantly associated with TMEM219 but not with IGFBP-3 expression. Higher TMEM219 expression was associated with better survival in bladder urothelial carcinoma (FDR = 9.54∙10^−3^ (HR = 0.61)), while the reverse was true for head and neck squamous cell carcinoma (FDR = 4.17∙10^−2^ (HR = 1.43)). On the other hand, the expression of TMEM219 was not significantly associated with survival in breast cancer, while IGFBP-3 expression was positively associated with survival outcome (Figure 8). These results indicate that the effect of TMEM219 expression on survival is less pronounced and highly cancer-specific.

### 4.3. Survival Effect of IGFBP-3 and TMEM219 in Clinical Subcategories

By taking advantage of the availability of clinical annotations, survival analysis of the effect of IGFBP-3 and TMEM219 expression in clinical subgroups, e.g., “race-black or African-American” was further performed. Similar to all cancer analyses, *p*-values were corrected for multiple testing across all tested subgroups in a given cancer. The advantage of such analyses is that they provide detailed insights into the effect of IGFBP-3 and TMEM219 in different subgroups of patients. The disadvantage is that some subgroups have an insufficient number of patients, e.g., in the “race-black or African-American” subgroup, which limits the cross-cancer comparisons.

Given the high significance of IGFBP-3 gene expression on survival outcome in glioma (FDR = 1.51∙10^−32^ (HR = 4.39)), it was unsurprising that IGFBP-3 expression affected survival in nearly all glioma subgroups (FDR < 2.90∙10^−2^), with lower expression being associated with better survival outcome. Similarly, all subgroups in lower grade glioma were significantly associated with IGFBP-3 expression, with lower expression indicative of better survival outcome (FDR < 5.82∙10^−2^). Similar results were observed for clinical subgroups in mesothelioma, the pan-kidney cohort, rectum adenocarcinoma, colorectal adenocarcinoma, and colon adenocarcinoma cancers, where low expression of IGFBP-3 was similarly associated with better survival outcome. These results confirm previous observations that the expression of IGFBP-3 may affect survival in glioma, mesothelioma, kidney, and colorectal cancers.

**Figure 9:**
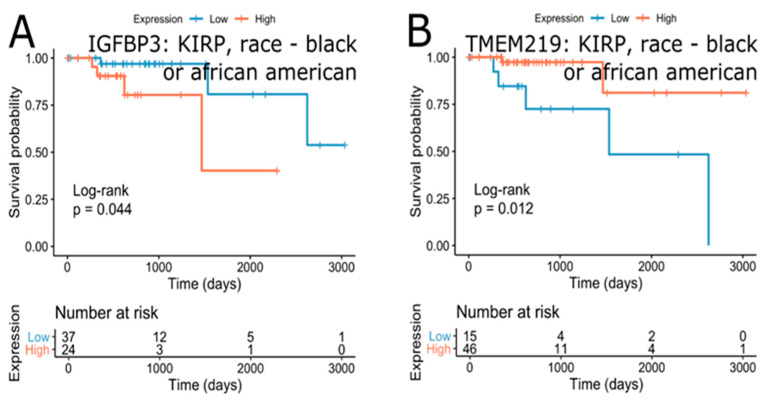
Survival effect of IGFBP3 (**A**) and TMEM219 (**B**) in “race-black or African-American.” Kidney renal papillary cell carcinoma. Unadjusted p-values are shown.

Further analyses of the effect of IGFBP-3 and TMEM219 expression in specific clinical subgroups revealed that kidney renal papillary cell carcinoma is the only cancer where the expression of both IGFBP-3 and TMEM219 is marginally associated with survival in “race-black or the African-American” subgroup. Lower IGFBP-3 expression was beneficial for survival (FDR = 5.84∙10^−2^ (HR = 5.12)), while higher TMEM219 expression was associated with better survival in the “race-black or African-American” subgroup (FDR = 6.28∙10^−2^ (HR = 0.16)), Figure 9). These results suggest the importance of the IGFBP-3/TMEM219 system in kidney renal papillary cell carcinoma in the “race-black or African-American” subgroup.

### 4.4. Survival Effect of IGFBP-3 and TMEM219 in Breast Cancer

**Figure 10:**
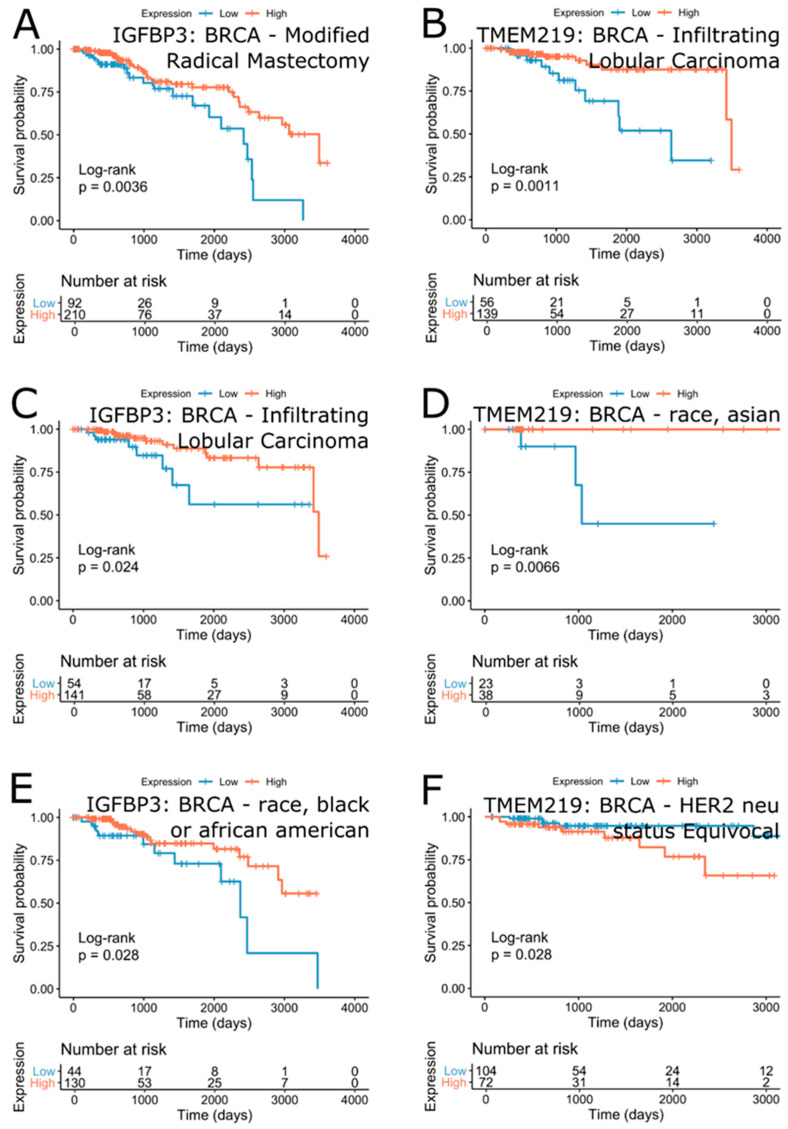
Survival effect of IGFBP-3 (**A**,**C**,**E**) and TMEM219 (**B**,**D**,**F**) in selected breast cancer subgroups. Unadjusted *p*-values are shown.

Since the expression of IGFBP-3 and TMEM219 was not significantly associated with survival outcome in breast cancer, it is possible that heterogeneity of the disease may prevent the detection of significant associations. Consequent analysis of the survival effect of IGFBP-3 and TMEM219 in clinical subgroups of the breast cancer cohort revealed that clinical subgroup annotated as “histological type-Infiltrating Lobular Carcinoma” show marginally significant association of IGFBP-3 (FDR = 2.01∙10^−1^ (HR = 0.36)) and TMEM219 ((3.96∙10^−2^ (HR = 0.25)) with the survival outcome (Figure 10B,C). For both genes, high expression was associated with a better prognosis. These results suggest that targeting the IGFBP-3/TMEM219 system in patients diagnosed with infiltrating lobular carcinoma, which is the second most common type of breast cancer, may be beneficial. Other clinical subgroups of breast cancer patients included “breast_carcinoma_surgical_procedure_name-Modified Radical Mastectomy” (IGFBP-3, FDR = 1.26∙10^−1^ (HR = 0.44), Figure 10A), “lab_proc_her2_neu_immunohistochemistry_receptor_status-Equivocal” (TMEM219, FDR = 3.27∙10^−1^ (HR = 3.29)). Of note were race-specific survival effects with high expression of IGFBP-3 being beneficial in the “race-black or African-American” subgroup (FDR = 2.01∙10^−1^ (HR = 0.42), Figure 10E) and TMEM219 high expression being beneficial in the “race-Asian” subgroup (FDR = 1.16∙10^−1^ (HR = 0.00), Figure 10D). Confirming our previous observations, the survival benefits of IGFBP-3 expression in breast cancer were consistently associated with high IGFBP-3 expression, while the effect of TMEM219 was more diverse and subgroup-specific.

## 5. Conclusions

IGFBP-3 is a multifunctional protein and is involved in the pathophysiology of a variety of human diseases such as cancer, diabetes, fatty liver disease, ischemia, and Alzheimer’s disease. Apart from the IGF/IGF-IR-dependent actions, IGFBP-3 exerts multiple biological activities through the IGF/IGF-IR-independent actions by interacting with distinct interacting proteins on the cell surface or within the cell. Much attention was given to identify a putative receptor for IGFBP-3 since early studies have demonstrated the anti-tumor function of IGFBP-3 in cancer. As described in this review, a few membrane proteins have been identified as “a putative IGFBP-3 receptor” and further characterized their functions with potential underlying mechanisms in cancer cells. Among them, TMEM219 appears to be the most critical IGFBP-3 receptor mediating anti-tumor and anti-metastatic activities of IGFBP-3. Given the fact that IGFBP-3/IGFBP-3R (TMEM219) axis is impaired and shown to have great impact on the survival outcome in specific cancers, IGFBP-3 and TMEM219 may serve as new diagnostic and prognostic biomarkers in specific cancers. Importantly, IGFBP-3R (TMEM219) agonists, in particular TMEM219 agonistic mAbs, are very attractive cancer therapeutics since these agonists would exhibit no other biological activities of IGFBP-3 induced by the interaction with other binding partners. Further characterization of specific gene regulation by TMEM219 activation and its crosstalk with other key signaling pathways will open a new avenue to treat many different types of cancer as a targeted monotherapy and a combination therapy with other chemotherapies.

## Figures and Tables

**Table 1:** Differential expression of IGFBP-3. “Mean log2(RSEM)”—average gene expression in normal and cancer tissues, respectively. *t*-test *p*-values are shown.

TCGA ID	Description	log2 Fold Change	*p*-Value	Mean log2(RSEM) Normal	Mean log2(RSEM) Cancer
**Upregulated in Tumor**					
KIRC	Kidney renal clear cell carcinoma	3.44	3.11 10^−53^	11.86	15.30
LUSC	Lung squamous cell carcinoma	2.26	1.22 10^−28^	10.61	12.86
LUAD	Lung adenocarcinoma	1.94	9.28 10^−21^	10.31	12.26
HNSC	Head and Neck squamous cell carcinoma	1.32	2.29 10^−7^	10.82	12.14
STAD	Stomach adenocarcinoma	1.18	8.67 10^−7^	10.82	12.00
THCA	Thyroid carcinoma	1.11	4.85 10^−16^	9.46	10.57
BLCA	Bladder urothelial carcinoma	1.05	3.57 10^−3^	12.56	13.61
COAD	Colon adenocarcinoma	0.65	4.01 10^−9^	10.45	11.10
KIRP	Kidney renal papillary cell carcinoma	0.34	5.19 10^−2^	11.69	12.03
**Downregulated in Tumor**					
LIHC	Liver hepatocellular carcinoma	−2.53	4.17 10^−81^	14.18	11.65
KICH	Kidney Chromophobe	−1.07	6.80 10^−5^	12.32	11.25
BRCA	Breast invasive carcinoma	−0.82	9.74 10^−24^	12.04	11.23
PRAD	Prostate adenocarcinoma	−0.56	1.47 10^−5^	11.27	10.72
UCEC	Uterine Corpus Endometrial Carcinoma	−0.43	1.53 10^−1^	13.29	12.86

**Table 2:** Differential expression of TMEM219. “Mean log2 (RSEM)”—average gene expression in normal and cancer tissues, respectively. *t*-test *p*-values are shown.

TCGA ID	Description	log2 Fold Change	*p*-Value	Mean log2(RSEM) Normal	Mean log2(RSEM) Cancer
**Upregulated in Tumor**					
KIRP	Kidney renal papillary cell carcinoma	0.53	3.16 10^−14^	11.07	11.60
THCA	Thyroid carcinoma	0.40	2.77 10^−8^	10.94	11.34
BRCA	Breast invasive carcinoma	0.39	1.00 10^−19^	10.71	11.10
KIRC	Kidney renal clear cell carcinoma	0.29	4.30 10^−11^	10.88	11.18
BLCA	Bladder urothelial carcinoma	0.27	1.08 10^−1^	10.92	11.19
UCEC	Uterine Corpus Endometrial Carcinoma	0.22	4.47 10^−3^	11.10	11.32
LIHC	Liver hepatocellular carcinoma	0.09	2.11 10^−1^	11.30	11.39
PRAD	Prostate adenocarcinoma	0.08	2.52 10^−1^	11.18	11.26
**Downregulated in Tumor**					
LUSC	Lung squamous cell carcinoma	−0.74	6.56 10^−23^	11.18	10.45
STAD	Stomach adenocarcinoma	−0.42	5.93 10^−4^	10.86	10.44
COAD	Colon adenocarcinoma	−0.36	1.47 10^−7^	11.25	10.89
HNSC	Head and neck squamous cell carcinoma	−0.32	9.00 10^−4^	10.63	10.30
LUAD	Lung adenocarcinoma	−0.12	3.97 10^−2^	11.18	11.06
KICH	Kidney Chromophobe	−0.10	3.20 10^−1^	11.24	11.14
